# Retraction: Boswellic Acid Suppresses Growth and Metastasis of Human Pancreatic Tumors in an Orthotopic Nude Mouse Model through Modulation of Multiple Targets

**DOI:** 10.1371/journal.pone.0275582

**Published:** 2022-09-28

**Authors:** The PLOS ONE Editors

After this article [1] was published, concerns were raised about the mouse tumor sizes reported in Figure 2. Specifically:

- The chart in Figure 2B of the article [1] reports tumour sizes of up to 5500 Photons/cm^2^/sec/steradian, with a standard error of approximately 1500 Photons/cm^2^/sec/steradian.
- The chart in Figure 2D of the article [1] reports tumour sizes of up to 23 cm^3^, with a standard error of approximately 6 cm^3^.

The journal asked the corresponding author to provide the individual-level data underlying these results and information about humane endpoints and health monitoring during the tumour experiments. In response, the corresponding author stated that they no longer have access to the data or the requested information.

*PLOS ONE* Editors consulted an expert in animal welfare and animal research methodology about this case. The expert assessed the article and noted that since these tumours are implanted orthotopically and not subcutaneously, typical tumour size limits for subcutaneous humane endpoints do not apply. However, they also stated that weekly monitoring of tumour volume as reported in the article would not have met U.S. standards for mouse tumour studies.

A representative of the Institutional Animal Care and Use Committee (IACUC) at The University of Texas MD Anderson Cancer Center also reviewed this paper in comparison to the animal research protocol approved by the IACUC in 2009 for this study (10-05-11032). They stated that what is reported in the article [1] is not in compliance with the approved IACUC protocol. The IACUC therefore recommended retraction of the article.

In light of the above concerns and the information received from the IACUC representative, *PLOS ONE* Editors concluded that the study did not comply with the journal’s Animal Research Policy. Therefore, the *PLOS ONE* Editors retract this article. The editors regret that these concerns were not identified prior to the article’s publication.

BP, SP, VY and BS did not respond or could not be reached. BBA did not respond to the retraction decision.
